# Uterine Torsion in an Elderly Woman Associated with Leiomyoma and Continuously Elevating Muscle Enzymes: A Case Study and Review of Literature

**DOI:** 10.1155/2020/8857300

**Published:** 2020-10-20

**Authors:** Hiroko Oda, Youko Yamada, Yuriko Uehara, Tamami Ohno, Mari Hoya, Mariko Sassa, Misako Mishima

**Affiliations:** Department of Obstetrics and Gynecology, Kawakita General Hospital, Tokyo, Japan

## Abstract

Uterine torsion is extremely rare in postmenopausal women. Total ischemia of the uterus may cause life-threatening conditions; hence, accurate diagnosis and surgical intervention are crucial. However, preoperative diagnosis is often challenging due to nonspecific clinical features and laboratory findings. We report a case of uterine torsion in a 73-year-old woman who presented with mild but gradually worsening intermittent abdominal pain. During a 5-day observation, repeated blood exams showed elevating serum muscle enzyme levels, lactate dehydrogenase (LDH), and creatinine kinase (CPK), in addition to nonspecific signs of inflammation. Computed tomography (CT) scans were obtained before and after the worsening of symptoms, which revealed changes in size and position of the enlarged uterus with a large leiomyoma, even within a 5-day interval. Based on these findings, the preoperative diagnosis was uterine torsion. Emergency surgery revealed a 540-degree torsion of the uterus at the cervix and uterine body junction. Total hysterectomy and bilateral salpingo-oophorectomy were performed. Plasma muscle enzyme levels normalized after surgery, and the patient recovered without complications. In conclusion, uterine torsion should be considered during differential diagnosis in elderly women with large leiomyoma, even when symptoms are mild. Elevating plasma muscle enzymes may be an indication of uterine torsion; hence, repeated laboratory works and CT scanning should be performed when symptoms progress. Comparison of CT images, taken before and after the worsening of symptoms, may also be relevant for diagnosis. Since uterine torsion may cause rapid deterioration and become life-threatening, early diagnosis and surgical intervention are crucial to avoid serious complications.

## 1. Introduction

Uterine torsion is an extremely rare condition that requires emergency surgery. It is defined as a rotation > 45 degrees around the long axis of the uterine body, characterized by various symptoms such as abdominal pain, vaginal bleeding, nausea, and vomiting [1–3] . Uterine torsion is reported in female patients of all ages, ranging from childhood to the postmenopausal period. Most cases are known to involve gravid uterus, large leiomyoma, or ovarian cyst [4, 5]. Uterine torsions in elderly women are rare and difficult to diagnose because they tend to present with vague symptoms and nonspecific laboratory findings [6]. Clinical findings or biomarkers specific for uterine torsion are unclear; thus, preoperative diagnosis is challenging, which can lead to the delay of surgical intervention. We report a case of a 73-year-old woman who was diagnosed preoperatively with uterine torsion by detecting continuously elevating plasma muscle enzyme levels and alterations in CT images that were taken repeatedly during the progression of symptoms.

## 2. Case Presentation

A 73-year-old nulligravid woman presented to our emergency room with severe abdominal pain. The patient recurrently experienced mild abdominal pain for the past week. She was examined at a nearby hospital 5 days before where plain CT scan and blood exams were performed. Her primary diagnosis was traction pain caused by a large uterine leiomyoma. Since her symptoms subsided with pain control medications, she was discharged from the hospital after 4 days of observation. However, she was referred to our facility the next day with complaints of a worsening abdominal pain. The patient had a medical history of an untreated large uterine leiomyoma diagnosed 25 years ago. She had stopped visiting her gynecologist after menopause at the age of 55. Although she felt a growing mass in her abdomen, it had been asymptomatic until the current episode, except for the sudden appearance of an umbilical hernia a few years ago. On admission, the patient was generally stable with normal vital signs. The abdomen was soft with no rebound tenderness or rigidity; however, a large mass in the left lower quadrant and an umbilical hernia were noted. Pain was localized over the abdominal mass, which was approximately the size of a second-trimester pregnancy and was easily movable by palpation. No vaginal bleeding was observed. Ultrasound imaging showed an abdominal mass > 10 cm in size and free fluid in the abdominal cavity. Blood exams (Table 1) indicated signs of inflammation with elevated white blood cell (WBC) count (21500/*μ*L) and C-reactive protein (CRP) levels (13.6 mg/dL), in addition to low hemoglobin levels (10.6 g/dL). Biochemistry tests showed elevated muscle enzyme levels, lactate dehydrogenase (LDH; 503 IU/L), and creatinine kinase (CPK; 601 IU/L). However, other parameters were within normal limits. Compared with the blood exams performed 5 days before, WBC, CRP, LDH, and CPK levels were significantly elevated. The level of hemoglobin had slightly decreased and mild coagulopathy was observed; however, it did not meet the diagnostic criteria for disseminated intravascular coagulation (DIC).

**Figure 1:**
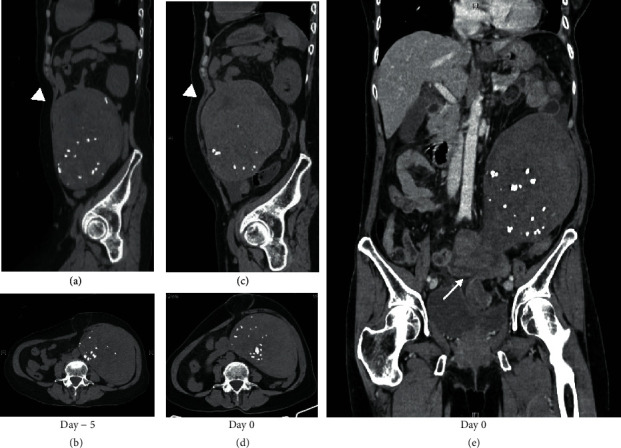
Computed tomography (CT) images of the uterus and leiomyoma. Comparison of sagittal (a, c) and axial (b, d) views of the plain abdominal CT images. Compared to the CT scan taken 5 days before the surgery (a, b), the leiomyoma had slightly enlarged in size and anteriorly deviated (white arrowheads) on the day of emergency surgery (c, d). The enhanced CT image (e) showed a twisted cervix at the level of the uterine body and cervical junction (white arrow).

**Figure 2:**
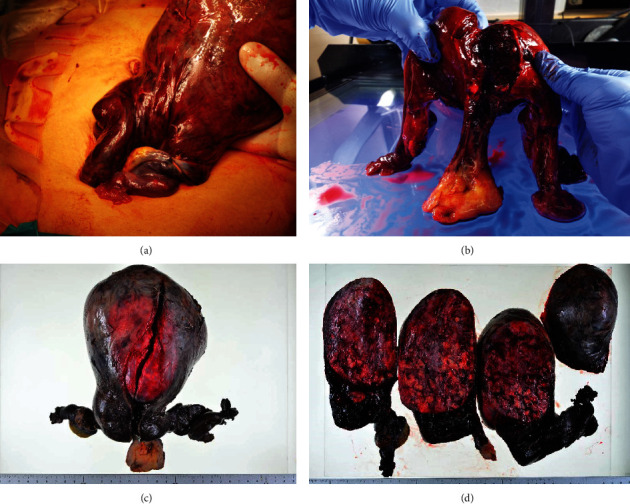
Operative findings and gross section of the uterus. The gross material revealed that the uterus was rotated 540 degrees counterclockwise at the level of the junction between the cervix and the uterine body. Both fallopian tubes and uterine body were discolored and necrotic. The cervix was atrophic and unusually elongated (a, b). Pathologic findings were uterine interstitial leiomyoma with diffuse calcification and hemorrhagic necrosis, weighing 1360 g in total (c, d). Both ovaries, fallopian tubes, and uterine body were necrotic, suggesting total tissue infarction due to the axial rotation of the uterus.

The patient underwent a plain CT scan, which revealed a large uterine leiomyoma with calcification measuring 15 × 13 × 9 cm (Figure 1(b)). Compared to the CT scan taken 5 days before, the leiomyoma that was previously 14 × 9 × 7 cm had slightly enlarged and deviated anteriorly, from the pouch of Douglas to the abdominal cavity. Diffuse high-density area was observed in the uterine body with an increasing amount of abdominal free fluid, indicating hemorrhagic tissue necrosis. Given these findings, contrast enhanced CT scan was additionally performed for a detailed evaluation. Enhanced CT images showed an elongated cervix, twisted at the uterine body and cervical junction (Figure 1(c)). The enhancement effect was diminished above the twisted cervix, including the whole uterine body and the leiomyoma. Our preoperative diagnosis was uterine torsion. The decision to perform an emergency surgery was made on the day of admission. Upon entry to the peritoneal cavity, 270 mL of hemorrhagic fluid was extracted. The uterus with a large leiomyoma was rotated 540 degrees counterclockwise at the level of the junction between the cervix and uterine body (Figures 2(a) and 2(b)). Both fallopian tubes and the uterine body were discolored and necrotic, indicating total tissue ischemia. The untwisted cervix was found to be atrophic and unusually elongated. Total hysterectomy, bilateral salpingo-oophorectomy, and umbilical hernia repair surgery were performed. The patient remained stable without blood transfusion, and her postoperative course was uneventful. Elevated plasma LDH levels and signs of inflammation improved on postoperative day 1. She was discharged on postoperative day 7 and has been followed up in our clinic. No postoperative complications are found so far. The final pathologic finding of the specimen was uterine interstitial leiomyoma with diffuse calcification and hemorrhagic necrosis, weighing 1360 g (Figures 1(c) and 1(d)). Both ovaries, fallopian tubes, and uterine body were necrotic, suggesting total tissue infarction due to the axial rotation of the uterus. No signs of malignancy were observed.

## 3. Discussion

We report a case of uterine torsion in a postmenopausal elderly woman, whose preoperative diagnosis was made by detecting elevating plasma muscle enzyme levels, LDH and CPK, and alterations in repeated CT images.

Clinical features of uterine torsion are usually nonspecific, varying from mild distention to severe abdominal pain with peritoneal signs or hemorrhagic shock. Although the condition could rapidly progress and become life-threatening, preoperative diagnosis is often difficult due to few known uterine-torsion-specific findings.

Typically, laboratory workups in patients with uterine torsion show signs of nonspecific inflammation, followed by progressive anemia and coagulopathy. Since biomarkers that specifically indicate uterine torsion are not known, blood exam results are not considered as key information for definitive diagnosis. In our current case, muscle enzyme levels, LDH and CPK, were gradually elevating after the onset of symptoms. Although it has not been reported in humans, the elevation of plasma muscle enzyme levels is frequently observed in bovine uterine torsion cases due to the damage of uterine muscles [7]. Clinically, elevating plasma LDH levels in patients with leiomyoma indicate hemorrhage or degeneration of the tumor, which is often a long-term change observed in cases of malignant sarcoma [8]. However, serum LDH and CPK levels were within the normal range in our patient's primary blood exams. Repeated blood exams showed an acute elevation in these muscle enzyme levels as the symptoms gradually worsened. It is possible that plasma LDH and CPK levels may have reflected the severity of muscle tissue damage due to uterine torsion. Our findings suggest that plasma muscle enzyme levels, such as LDH and CPK, may be a diagnostic biomarker of this condition.

CT scans are beneficial for the diagnosis of uterine torsion, especially when comparing images taken on different time points during the progression of symptoms. Generally, enhanced CT scans are highly recommended for the diagnosis of uterine torsion. A twisted cervix, known as the “whorled sign,” shown by contrast-enhanced CT scan is one of the characteristic findings [4]. However, enhanced CT scans are not always suitable for elderly patients, especially in those with a medical history of kidney failure and those exhibiting a potential risk of renal damage. In such cases, comparing past and present images of plain CT may be a useful diagnostic method. The growth of leiomyomas depends on estrogen; thus, they rarely increase in size after menopause. Leiomyomas suddenly enlarging in a postmenopausal woman may be an indication of acute hemorrhage or degeneration caused by uterine torsion. Wang et al. reported that three-dimensional reconstruction CT images are useful to detect the deviation of the uterus by tracking the movement of calcifications in leiomyoma [6]. Since our patient underwent CT scans repeatedly after the mild onset of symptoms, we were able to compare the size and position of the uterus that had changed even within a 5-day interval (Figure 1). The comparison of CT images from different time points may improve the early detection of uterine torsion in elderly patients.

By searching the literature, we found seven cases of uterine torsion in postmenopausal women reported in the last 10 years (2010–2020) (Table 2). Uterine torsion is most commonly reported during pregnancy, presenting with acute symptoms such as sharp abdominal pain, rigidity, and rebound tenderness [5]. Although very few cases have been reported, postmenopausal elderly patients tend to present with chronic mild symptoms in the early stage and deteriorate rapidly once their condition starts to progress. Large leiomyoma is a well-known risk factor of uterine torsion in nongravid women. All reported cases of uterine torsion in elderly patients over the age of 60 years involved leiomyoma larger than approximately 15 cm as a maximum diameter [2, 3, 6, 9, 10]. Interestingly, previous literature demonstrated that four out of six elderly patients, including our current case, presented with a medical history of hernia before the occurrence of uterine torsion [3, 6, 9]. Normally, the uterus is firmly stabilized and attached to the pelvic cavity by pelvic ligaments. In addition to the years of traction by heavy leiomyomas, the atrophic weakening of the pelvic ligaments, owing to the loss of collagen and elastin as patients get older, may contribute to their abnormal stretching. This may affect the stability of the uterus, causing its total rotation. Hernia is also an aging-related condition that occurs when the abdominal wall ligaments are weakened. Although not directly related, hernia could be a sign of weakening abdominal ligaments that could not tolerate the increased abdominal-cavity pressure caused by a large leiomyoma. A history of hernia could be another indicative factor of uterine torsion in elderly women.

As in our current case, clinical symptoms that could be decisive factors for surgical intervention, such as intense abdominal pain, rigidity, and rebound tenderness, were frequently absent in elderly patients aged 60 years and over [2, 3, 6] [9, 10]. Since the onset of symptoms is often unclear, most patients were observed for 5–7 days until surgical intervention was decided with or without preoperative diagnosis. In two reported cases of uterine torsion, patients, both below 60 years of age, presented with severe abdominal pain and underwent emergency surgery on the day of symptom onset [11, 12]. They were diagnosed with torsion of a large ovarian cyst, which might have been the main cause of pain, rather than an ischemic uterus. Clinical presentations may not necessarily reflect the severity of the uterus tissue ischemia. Even in cases with tightly twisted cervix of over 360 degrees, resulting in total blockage of the uterine blood flow, patients' chief complaint was mostly mild abdominal pain [6, 9, 10]. In some cases, surgical interventions were not decided until the patients' condition started to suddenly deteriorate due to massive abdominal hemorrhage. Intensive care including blood transfusion was required for such patients [2, 3].

According to these findings, we emphasize that it is crucial to consider uterine torsion during differential diagnosis in elderly women with a leiomyoma > 15 cm in diameter, even when abdominal symptoms are mild. Since elderly patients deteriorate rapidly due to irreversible uterine ischemia leading to coagulopathy and massive abdominal hemorrhage, early surgical intervention is critical to avoid serious complications.

## 4. Conclusion

Uterine torsion is an extremely rare condition in elderly women and is difficult to diagnose due to its unclear symptoms. Elevation of plasma muscle enzyme levels accompanied by signs of inflammation could be an indication of uterine torsion in women with large leiomyomas. Comparison of the CT images taken before and after the worsening of symptoms may also be relevant for diagnosis. The findings presented here imply that repeated laboratory workups and CT scanning should be considered when symptoms progress rapidly. Comparing the results may lead to early diagnosis and surgical intervention, which subsequently may prevent critical deterioration, thus improving the morbidity and mortality of patients.

## Figures and Tables

**Table 1:** Laboratory findings.

Elements	Day − 5	Day − 4	Day 0 operation	Day + 1	Reference
LDH (U/L)	198	196	503	207	106-220
CPK (U/L)	109	187	601	—	45-165
CRP (mg/dL)	0.06	0.1	13.6	25.9	0-0.3
WBC (cell/*μ*L)	10200	9000	21500	11600	4500-11000
Hb (g/dL)	14.3	12.8	10.6	7.6	11.0-14.6
Plt (10^4^ cell/*μ*L)	26.2	22.3	19.7	15.1	15.0-35
% PT (%)	106	—	71.5	—	80-100
PT INR	0.97	—	1.18	—	0.85-1.15

**Table 2:** Literature analysis on postmenopausal uterine torsion (PubMed available articles from 2010 to 2020).

Authors	Age	Symptoms	Peritoneal signs	Medical history	Time from onset to surgery	Torsion degree	Operation	Postoperative course
Halassy and Clarke [2]	70	Mild abdominal pain Back and shoulder pain	(-)	Leiomyoma (16 cm) Atrial fibrillation Type 2 DM	2 days	180	ATH+BSO	Blood transfusion
Wang et al. [6]	86	Renal failure Abdominal discomfort Appetite loss and delirium	(-)	Leiomyoma (16 cm) Umbilical hernia	>7 days	360	ATH+BSO	
Chua et al. [10]	73	Mild abdominal pain Back pain	(-)	Leiomyoma (18 cm)	14 days	360	ATH+BSO	
Yap et al. [12]	57	Severe abdominal pain Nausea	Unknown	Ovarian cyst (31 cm)	1 day	180	ATH+BSO	
Havaldar and Ashok [11]	55	Severe abdominal pain Nausea and distention	(+)	Ovarian cyst (21 cm)	1 day	180	ATH+BSO	
Sikora-Szczesniak et al. [3]	67	Mild abdominal pain	(-)	Leiomyoma (27 cm) Periumbilical hernia	5 days	180	AHT+BSO	Blood transfusion
Luk et al. [9]	61	Mild abdominal pain Distention	(-)	Leiomyoma (15.5 cm) Hiatus hernia	5 days	720	ATH+BSO	Hemorrhagic shock

ATH: abdominal total hysterectomy; BSO: bilateral salpingo-oophorectomy. Maximum diameters of the leiomyomas or ovarian cysts are enclosed in parentheses.

## Data Availability

The data used to support the findings of this study are available from the corresponding author upon request.
